# The Role of Plant Evolutionary History in Shaping the Variation in Specific Leaf Area Across China

**DOI:** 10.1002/ece3.71304

**Published:** 2025-04-18

**Authors:** Minyue Si, Caiyi Zhang, Chunzhu Xiang, Mingxia Jiang, Linwei Guo, Junjiong Shao

**Affiliations:** ^1^ National key Laboratory for Development and Utilization of Forest Food Resources, College of Forestry and Biotechnology Zhejiang A&F University Hangzhou China; ^2^ Tianmushan Forest Ecosystem National Orientation Observation and Research Station of Zhejiang Province Hangzhou China

**Keywords:** angiosperms, evolutionary history, gymnosperms, phylogenetic eigenvector regression, phylogenetic signals, relative contribution

## Abstract

Specific leaf area (SLA, leaf area per unit leaf dry mass) occupies a central position in both community assembly and ecosystem functioning. Although SLA has significant phylogenetic signals, how and to what extent the evolutionary history influences the variation in SLA remain poorly understood. In this study, based on a dataset containing 1264 plant species belonging to 549 genera and 141 families in gymnosperms, monocots, and eudicots across China, we analyzed the influences of climatic conditions and soil properties on SLA, calculated the phylogenetic signals of SLA, and quantified the relative contributions of evolutionary history (represented by interspecific relatedness and intraspecific variation) to the variation in SLA. The results showed that the interspecific relatedness accounts for 50.46% of the total variance in SLA, followed by the intraspecific variation (36.12%), climatic conditions (30.68%), and soil properties (24.74%). Along the phylogenetic tree, the split between angiosperms and gymnosperms had the largest contribution to the variation in SLA. Other detailed splits (e.g., the split between monocots and eudicots, the splits within Rosidae, and etc.) had significant but much smaller contributions. The relationship between SLA and environmental variables (climatic conditions and soil properties) was different between angiosperms and gymnosperms, with the climatic conditions having larger influences on SLA than the soil properties, implying interactive effects between environment and evolutionary history on SLA. Within the woody angiosperms, deciduous and evergreen species exhibited differential responses of SLA to climatic and soil factors, suggesting a non‐negligible role of leaf longevity in explaining the variation in SLA. Our results highlighted a much more important role of evolutionary history in the variation in SLA than previous studies. Neglecting such a great contribution could lead to biased conclusions if the evolutionary rate does not keep pace with the rapidly changing environments in the future.

## Graphical abstract

In this study, we analyzed the influences of climatic conditions and soil properties on the specific leaf area (SLA), calculated the phylogenetic signals of SLA, and quantified the relative contributions of evolutionary history to the spatial variation in SLA. We underscored the importance of evolutionary history in shaping the spatial variation of SLA across China, with the relative importance being 50.46%, substantially larger than those of climate and soil conditions, and highlighted a much more important role of evolutionary history in the spatial variation of SLA than previous studies.

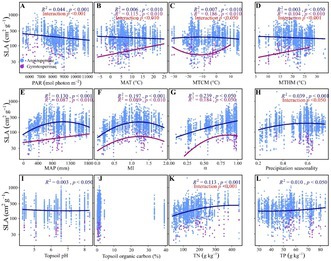

## 1 Introduction

Earth has recently experienced profound climate changes such as rising mean global temperature and shifting precipitation patterns (Masson‐Delmotte et al. 2021). This increases interest in research of plant and ecosystem responses to changing climate (Culshaw et al. 2021). As the core component of the leaf economics spectrum that indicates the resource‐use strategies from conservative to acquisitive, the specific leaf area (SLA, the ratio of leaf area to leaf dry mass, which is also the reciprocal of the leaf mass per area, LMA) is critical to community assembly and ecosystem functions (Loughnan and Gilbert 2017; Zirbel et al. 2017). For example, a larger SLA was associated with a smaller leaf mass fraction, which caused lower light inception and a weaker competitive effect (Kunstler et al. 2016). In a seasonally dry tropical forest, lower SLA indicated more efficient use of scarce water in old communities, implying a critical role of SLA in forest succession (Subedi et al. 2019). At the ecosystem level, SLA was not only strongly correlated to ecosystem productivity, evapotranspiration, and litter decomposition (Shao et al. 2019; An et al. 2021; Cornelissen et al. 2007) but also can regulate or moderate the effects of climate changes (e.g., CO_2_ enrichment, and global warming) on plant growth and ecosystem carbon sequestration (Kovenock and Swann 2018; Shao et al. 2019).

Large variation in SLA was found on the regional and global scales (e.g., 6.8–1136.4 cm^2^ g^−1^ on the global scale; Flores et al. 2014), usually along environmental gradients (Wright et al. 2004; Vandvik et al. 2020). For example, SLA decreased with the increase in altitude at the Amazon–Andes gradient in Peru (van de Weg et al. 2009). SLA shows positive or no correlation with latitude (Wang et al. 2016a; Luo et al. 2019). Even at finer scales, SLA could exhibit large variations. For example, in a temperate broad‐leaved mixed forest in China, the SLA of shade and herbaceous plants is larger than that of sun and woody plants, respectively (Liu, He, et al. 2022).

This variation in SLA can be driven by climatic conditions, soil properties, and plant characteristics (e.g., plant functional type, Wang et al. 2016a; Xing et al. 2021). The response of SLA to climatic drivers is inconsistent among different studies. For example, some demonstrated a negative correlation between SLA and mean annual temperature (MAT, Wright et al. 2004; Duan et al. 2022), whereas others reported positive associations (Liu, Wang, et al. 2017). For the temperate forests in China, SLA first increased and then decreased with the increase in MAT (Liu, Zhao, et al. 2022). Similarly, positive or negative correlations between SLA and mean annual precipitation (MAP) were found in different studies (Torres‐Ruiz et al. 2019; Liu, Zhao, et al. 2022). These inconsistent results might reflect the interactive effects of climatic variables (e.g., temperature‐precipitation covariation) and divergent species‐specific adaptive strategies across plant functional types (Wright et al. 2005; An et al. 2021).

Soil properties were another group of factors that can strongly influence SLA. Generally, SLA increased with soil fertility (Libalah et al. 2017). The positive correlation between SLA and soil nitrogen (N) availability was widely found (Ordoñez et al. 2009; Liu, Wang, et al. 2017). Higher N availability not only reduces the lignin content and increases the leaf protein concentration in plant leaves but also decreases the accumulation of nonstructural carbohydrates (TNC), resulting in lower cost of defense and higher allocation to leaf area (Poorter et al. 2009; Tang et al. 2021). In addition, SLA may decrease with soil pH and C:N but with differential rates of changes among herbs, shrubs, and trees (Gong and Gao 2019).

Besides climatic conditions and soil properties, plant characteristics, usually indicated by the plant functional types (PFTs), may also be critical to the SLA. For example, herbaceous species pursue a more resource‐acquisitive strategy than woody species do, resulting in a greater SLA (Wright et al. 2005; Rossatto and Franco 2017). Compared to deciduous woody plants, evergreen ones not only had lower SLA but also exhibited opposite trends of SLA with MAT and rainfall (Wright et al. 2005; Li et al. 2013). Broadleaf species have a greater SLA than coniferous species (Wang et al. 2016a). Within herbaceous plants, higher SLA was found in annuals than in perennials, which reflects that the annuals allocate more resources to leaves to support faster growth and complete the life cycle in a short period (Ning et al. 2022). At a much finer scale, leaf structural components, such as cell dry mass density, number of cell layers, and cell volume, were found to be the most important intrinsic drivers of SLA (John et al. 2017).

Despite the above‐mentioned progress, a critical factor, plant evolutionary history, has been largely neglected. Indeed, some studies found significant phylogenetic signals in SLA at the regional and global scales (Flores et al. 2014; Liu, Zhao, et al. 2022), but it is unclear to what extent the evolutionary history could contribute to the variation in SLA. During the long‐term evolutionary course, plants can adjust their functional traits to adapt to environmental fluctuations (Thuiller et al. 2011). It is suggested that closely related species tend to exhibit greater ecological similarity than would be expected solely from their phylogenetic relationships, which phenomenon is termed the phylogenetic niche conservatism (Losos 2008). Therefore, one of our objectives in this study is to evaluate how “conservative” the SLA is within the various plant lineages (especially the clades within angiosperm).

In this study, based on the dataset containing 1264 plant species across China, we analyzed the influences of climatic conditions and soil properties on SLA, calculated the phylogenetic signals of SLA, and quantified the relative contributions of plant evolutionary history to the spatial variation in SLA. We also explored the relative contribution of each phylogenetic split to the tree‐wide variation in SLA to deepen the understanding of the detailed roles that evolutionary history may play in regulating the trait variation across a large spatial scale.

## 2 Materials and Methods

### 2.1 Data Source

**FIGURE 1:**
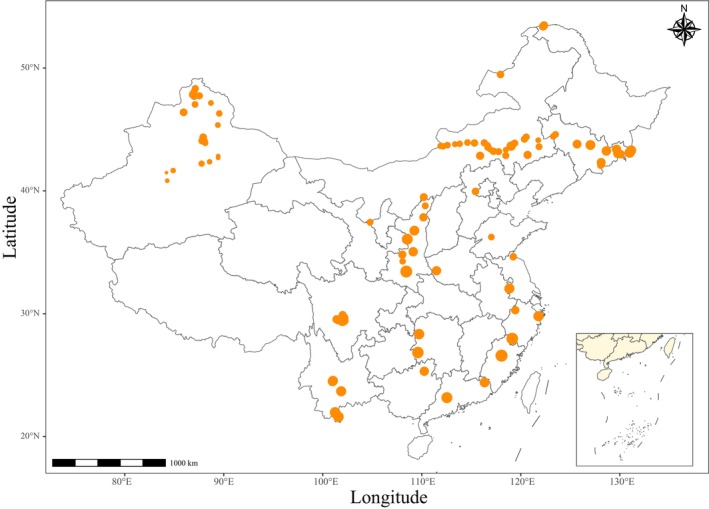
The sites with SLA observations in China. Point size represents the number of species in each site.

**FIGURE 2:**
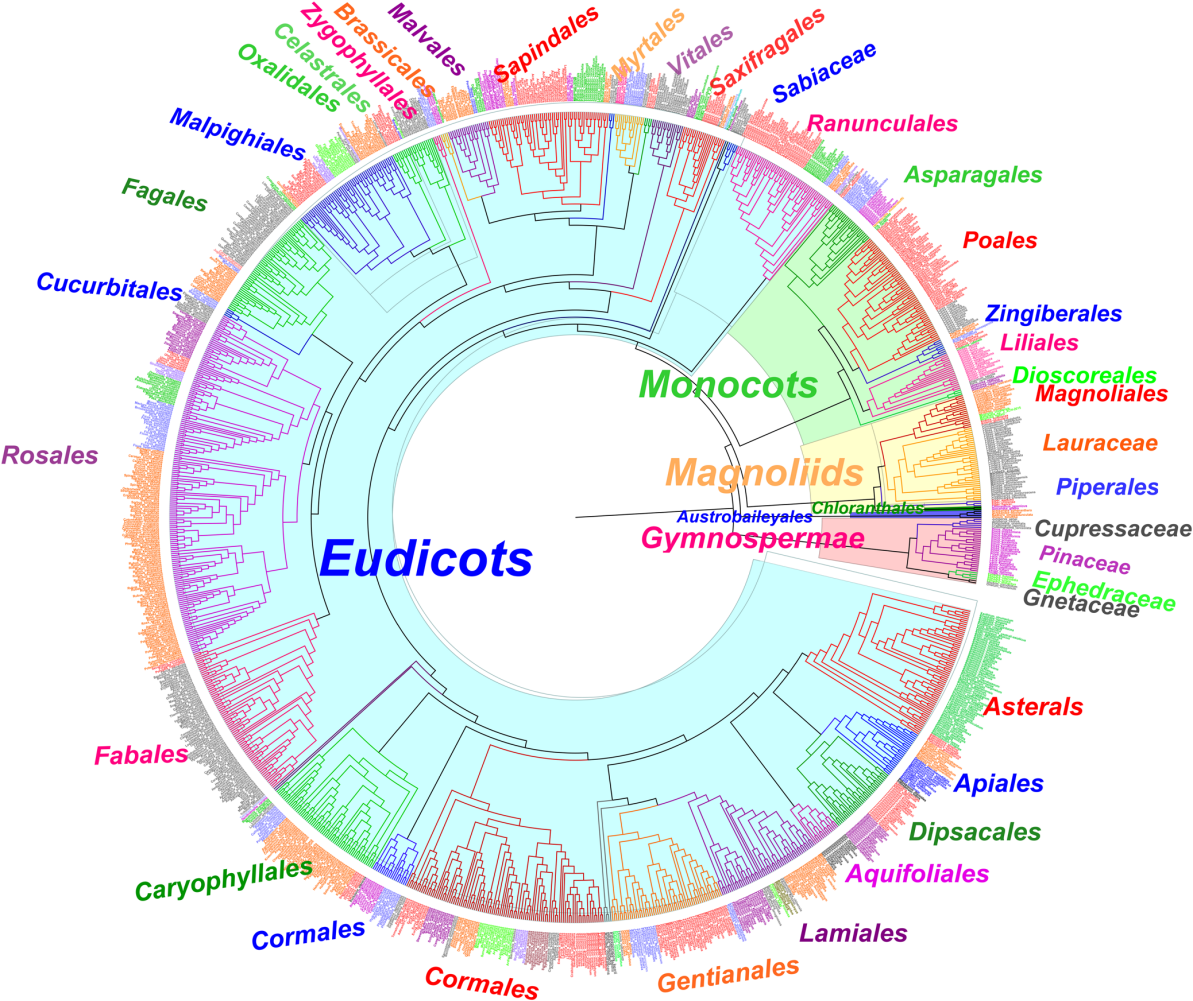
Phylogenetic tree of 1264 plant species in China. The species names in the same family are in the same color. The branches of the same order are in the same color. The orders with more than 5 species were labeled. Totally, there are 33 gymnosperms, 57 magnoliids, 117 monocots, and 1051 eudicots.

The data of the specific leaf area (SLA) and corresponding climatic conditions and soil properties were extracted from the China Plant Trait Database version 2 (CPTDv2; Wang et al. 2022). We excluded the data of ferns and bryophytes and of the plants without species names. As a result, there were a total of 2412 entries of 1264 spermatophyte species from 131 sites, which belong to 549 genera and 141 families in gymnosperms, monocots, and eudicots (Figures 1 and 2). The SLA values were log_10_‐transformed to achieve the normality assumption. The climate variables included annual photosynthetically active radiation (PAR), mean annual temperature (MAT), mean temperatures of the coldest and hottest months (MTCM, MTHM), mean annual precipitation (MAP), moisture index (MI, ratio of actual evapotranspiration to precipitation), ratio of actual to equilibrium evapotranspiration (*α*), and the seasonality of precipitation. The soil variables included the topsoil sand, silt, and clay fractions, pH, organic carbon, and cation exchange capacity. Additionally, the soil total nitrogen (TN) and phosphorus (TP) contents were extracted from the High Resolution National Soil Information Grids database of China from Soil SubCenter, National Earth System Science Data Center, National Science and Technology Infrastructure of China (Liu et al. 2020; Liu et al. 2021, http://www.geodata.cn).

The phylogenetic trees for the 1264 species were extracted from a mega‐tree (GBOTB.extended.TPL.tre) by using the R package *V.PHYLOMAKER2* (Jin and Qian 2022; Figure 2). The mega‐tree comprises 74,529 species of vascular plants in 10,597 genera and 482 families, based on The Plant List (TPL) nomenclature standardization system. The species names were standardized by TPL Nomenclature Standardization System, and the species that were absent from the mega‐tree were substituted with random species in the same genus.

### 2.2 Variance Decomposition

The total variance in SLA was decomposed into different spatial and taxonomic scales based on the nested linear random‐effects models. For the spatial scales, the random effects were the species nested within the sites. For the taxonomic scales, the random effects were the species nested within the genera, which in turn were nested within the families. This variance decomposition was calculated by the function *calcVarPart* in the R package *variancePartition* (Hoffman and Schadt 2016).

The total variance in SLA was also decomposed along the phylogenetic tree by the relative contribution index (CI) of each node to tree‐wide variation (Webb et al. 2011). CI measures the contribution of individual divergences to contemporary trait variation (Moles et al. 2005; Shao et al. 2019). This index was calculated by the function *ph_aot* in the R package *phylocomr* (Ooms et al. 2023).

### 2.3 Influences of Climatic Conditions and Soil Properties

The linear mixed‐effects models (LMMs) were used to investigate the relationships between the SLA and non‐phylogenetic variables (i.e., climatic conditions and soil properties). For a relationship between SLA and a specific independent variable (e.g., MAT), the independent variable was the fixed‐effect term, and the species identity was the random term. Models with linear and quadratic fixed effects were conducted. The model with the lower Akaike information criterion (AIC) was selected to describe the relationship between SLA and the independent variable. LMMs were conducted by the function *lmer* in the R package *lme4* (Bates et al. 2015). LMMs were also used to investigate the potential differential effects of non‐phylogenetic variables on SLA between angiosperms and gymnosperms and between evergreen (*n* = 624) and deciduous woody angiosperms (*n* = 990). The difference between evergreen and deciduous gymnosperms was not examined because of the extremely low sample size of deciduous gymnosperms (*n* = 11).

### 2.4 Phylogenetic Signals

Four indices of phylogenetic signal in SLA were tested: Moran's *I*, Abouheif's *C*_mean_, Blomberg's *K*, and Pagel's *λ*. Moran's *I* is an autocorrelation coefficient representing the relationship between the trait variation across taxa and the phylogeny (Münkemüller et al. 2012). The inverse of the patristic distances was used to represent the phylogenetic information. Abouheif's *C*_mean_ is also an autocorrelation index, which tests for serial independence on the basis of the sum of the successive squared trait deviations between neighboring species (Abouheif 1999). Greater deviations of Moran's *I* and Abouheif's *C*_mean_ from zero suggest a stronger influence of phylogeny on SLA (Münkemüller et al. 2012).

Blomberg's *K* is the ratio between the mean squared error of the tip trait deviated from the phylogenetic corrected mean and the mean squared error determined by the variance–covariance matrix of the phylogeny assuming Brownian Motion (BM, Blomberg et al. 2003). A *K* = 0 indicates no phylogenetic signal, a *K* = 1 suggests that the trait evolution follows the BM model, and a *K* > 1 indicates stronger similarities among species with close phylogenetic relatedness than expected under the Brownian process.

Pagel's *λ* scales the Brownian phylogenetic covariances down to the observed ones that may also be influenced by non‐phylogenetic factors (Pagel 1999; Münkemüller et al. 2012). *λ* = 0 indicates the independence of trait values on phylogeny, and *λ* = 1 indicates that the trait variation along the phylogenetic tree follows a BM evolution mode. All the phylogenetic signals were calculated by the function *phyloSignal* in the R package *phylosignal* (Keck et al. 2016).

### 2.5 Phylogenetic Eigenvector Regression

The relative importance of phylogenetic and nonphylogenetic factors to the spatial variations in SLA was quantified by the phylogenetic eigenvector regression based on multiple linear regression models (PVR; Desdevises et al. 2003; Shao et al. 2019). The explanatory variables were classified into three groups: the phylogenetic, climatic, and soil variables. We used the eigenvectors, which were extracted from the phylogenetic distance matrix of 1264 species by applying principal coordinate analysis, to represent the phylogenetic variables (Desdevises et al. 2003; Diniz‐Filho et al. 2014; Shao et al. 2019). The eigenvectors were calculated by function *PVRdecomp* in the R package *PVR* (Santos 2018). According to the results of LMMs, the climate variables included PAR, mean temperature of the coldest and hottest month, MAP, MI, and α. Similarly, the soil variables included topsoil sand, silt, and clay fractions, pH, TN, and TP.

The variance partitioning procedure followed Shao et al. (2019). Briefly, seven linear multiple regression models (2^3^–1) including the three classes of variables (phylogenetic, soil, and climatic) and their combinations were fitted. For models containing climatic or/and soil variables, the second‐order terms were included as potential factors if the LMM results showed that the quadratic relationships were better than the linear ones; otherwise, only the first‐order terms were considered as the potential factors. The most parsimonious version of each model was selected by AIC. On the basis of the *R* ^*2*^ of the seven most parsimonious models, it is possible to estimate the individual, combined, and class‐specific contributions of each variable and their combinations (Shao et al. 2019). Moreover, to investigate the potential influences of interactive effects between climatic and soil variables on the SLA, linear multiple regression models with interactive terms were also applied to divide the total variance in SLA into phylogenetic, climatic, and soil parts. All possible two‐way interactive terms were included in the models, and the AIC was used to select the best model.

The relative proportion of intraspecific variation was determined from the residuals of the model (model PCS) with all the three types of variables as explanatory variables. The residuals were used as the response variable and the species names as the explanatory variable in a linear regression model (model R). The relative importance of intraspecific variation was referring to (1−$R_{\mathrm{PCS}}^{2}$) (1−$R_{R}^{2}$), where 1−$R_{\mathrm{PCS}}^{2}$ and 1−$R_{R}^{2}$ are the unexplained proportions of variance by models PCS and R, respectively. Such a procedure was also used to investigate the relative contribution of evolutionary history to SLA between evergreen and deciduous woody angiosperms.

## 3 Results

### 3.1 Variation in Specific Leaf Area

**FIGURE 3:**
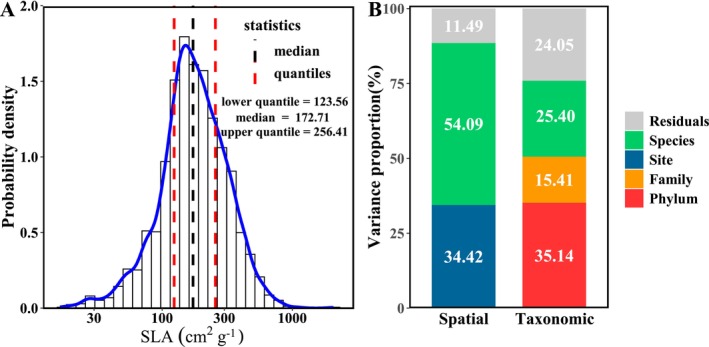
Histogram (A) and variance partitioning (B) of specific leaf area (SLA) across China. The blue line in (A) was the kernel density estimation of SLA distribution. The variance proportion in (B) is partitioned based on different spatial (left) or taxonomic scales (right) using nested linear random‐effects models.

The specific leaf area (SLA) across China showed a large variation (16.42 to 2056.92 cm^2^ g^−1^) with an average of 204.09 cm^2^ g^−1^ and a median of 256.41 cm^2^ g^−1^ (Figure 3A). Based on the spatial scale, the variation between species within the same site (54.09%) was larger than that among different sites (34.42%, Figure 3B). Based on the taxonomic scale, the phylum explained the most proportion of variation in SLA (35.14%), followed by the species (25.40%) and the family (15.41%, Figure 3B).

### 3.2 Differential Influences of Climatic Conditions and Soil Properties Between Angiosperms and Gymnosperms

**FIGURE 4:**
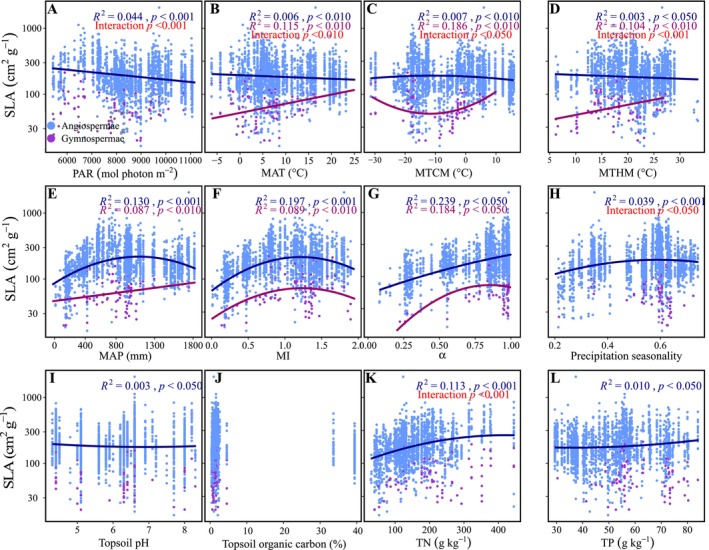
Influences of climatic conditions and soil properties on SLA of gymnosperms and angiosperms. Blue and purple colors represent the angiosperms and gymnosperms, respectively. Red text show the test result of the difference between angiosperms and gymnosperms. Regression lines are based on the linear mixed‐effects models. MAP, mean annual precipitation; MAT, mean annual temperature; MI, moisture index; PAR, annual photosynthetically active radiation; TN, soil total nitrogen content; TP, soil total phosphorus content; α, ratio of actual to equilibrium evapotranspiration.

The influences of climatic conditions and soil properties on SLA were different between angiosperms and gymnosperms. The SLA of angiosperms showed significant associations with PAR, precipitation seasonality, soil pH, soil N and P contents, and topsoil sand, silt, and clay fractions, while that of gymnosperms was not influenced by these factors (Figure 4A,H,I,K,L, Figure S1A–C). For the thermal factors, the SLA of angiosperms decreased with the increases in MAT, MTCM, and MTHM (*R* ^2^ = 0.003–0.007, *p* < 0.05; Figure 4B–D). The SLA of gymnosperms increased with the increases in MAT and MTHM and showed a nonlinear relationship with MTCM (*R* ^2^ = 0.104–0.186, *p* < 0.01; Figure 4B–D).

For the water factors, both angiosperms and gymnosperms showed nonlinear relationships between SLA and MI (*R* ^2^ = 0.197 and 0.089, respectively, both *p* < 0.01, Figure 4F). The SLA of angiosperms increased linearly with *α* (*R* ^2^ = 0.239, *p* < 0.05) but had a nonlinear relationship with MAP (*R* ^2^ = 0.130, *p* < 0.001), while that of gymnosperms increased linearly with MAT (*R* ^2^ = 0.087, *p* < 0.01) but had a nonlinear relationship with *α* (*R* ^2^ = 0.184, *p* < 0.05; Figure 4E–G).

### 3.3 Differential Influences of Climatic Conditions and Soil Properties Between Evergreen and Deciduous Woody Angiosperms

**FIGURE 5:**
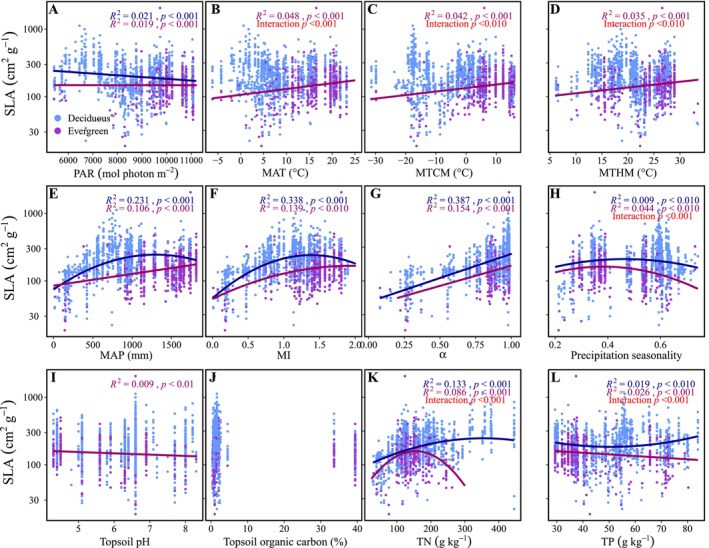
Influences of climatic conditions and soil properties on SLA of deciduous, evergreens in angiosperms. Blue and purple colors represent the deciduous and evergreens, respectively. Red text show the test result of the difference between deciduous and evergreens woody angiosperms. Regression lines are based on the linear mixed‐effects models.

Within the angiosperms, there were differential effects of climatic conditions and soil properties on SLA between evergreen and deciduous woody plants. With the increase in PAR, the SLA decreased in deciduous (*R* ^2^ = 0.021, *p* < 0.001) but increased in evergreens (*R* ^2^ = 0.019, *p* < 0.001; Figure 5A). The SLA of evergreens increased with the increases in MAT, MTCM, and MTHM (*R* ^2^ = 0.035–0.048, *p* < 0.001), while that of deciduous showed non‐significant trends (Figure 5B–D).

For the water factors, the SLA of deciduous first increased and then decreased with the increases in MAP, MI, and precipitation seasonality (*R* ^2^ = 0.009–0.338, *p* < 0.01) and increased with the increase in *α* (*R* ^2^ = 0.387, *p* < 0.001; Figure 5E–H). The SLA of evergreens increased with the increases in MAP, MI, and *α* (*R* ^2^ = 0.106–0.154, *p* < 0.01), but decreased with the increase in precipitation seasonality (*R* ^2^ = 0.044, *p* < 0.01; Figure 5E–H).

For soil properties, the SLA of deciduous first increased and then decreased with the increases in TN (*R* ^2^ = 0.140, *p* < 0.001) and decreased with the increases in TP (*R* ^2^ = 0.019, *p* < 0.001; Figure 5K,L). The SLA of evergreens decreased with the increases in soil pH (*R* ^2^ = 0.009, *p* < 0.01) and TP (*R* ^2^ = 0.086, *p* < 0.001) and first increased and then decreased with the increases in TN (*R* ^2^ = 0.026, *p* < 0.001; Figure 5I,K,L).

### 3.4 Relative Importance of Phylogenetic Information, Intraspecific Variation, Climatic Conditions, and Soil Properties

**FIGURE 6:**
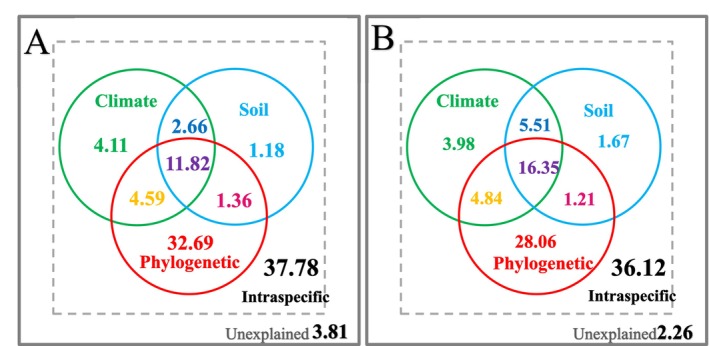
Relative contributions (%) of phylogenetic relatedness, intraspecific variation, climatic conditions, and soil properties to the variance in SLA based on the entire dataset. (A) Relative contributions derived from phylogenetic eigenvector regression (PVR) models without interactive effects. (B) Relative contributions derived from PVR with interactive effects between climatic and soil variables on SLA.

The phylogenetic signals for SLA were all significant, with the values of 0.0633, 0.3963, 0.0112, and 0.7371 for Moran's *I*, Abouheif's *C*_mean_, Blomberg's *K*, and Pagel's *λ*, respectively (Table 1). The PVR results were very similar between the models with and without interactive effects (Figure 6). Taking the models with interactive effects as examples, the majority of the variance in SLA (50.46%) was explained by phylogenetic relatedness, followed by intraspecific variation (36.12%), climatic conditions (30.68%), and soil properties (24.74%). The joint contributions of phylogenetic relatedness, soil properties, and climatic conditions were 16.35%.

**TABLE 1:** Phylogenetic signals of plant SLA across China.

Phylogenetic signal	Value	*p*
Moran's *I*	0.0633	0.001
Abouheif's *C*_mean_	0.3963	0.001
Blomberg's *K*	0.0112	0.001
Pagel's *λ*	0.7371	0.001

**FIGURE 7:**
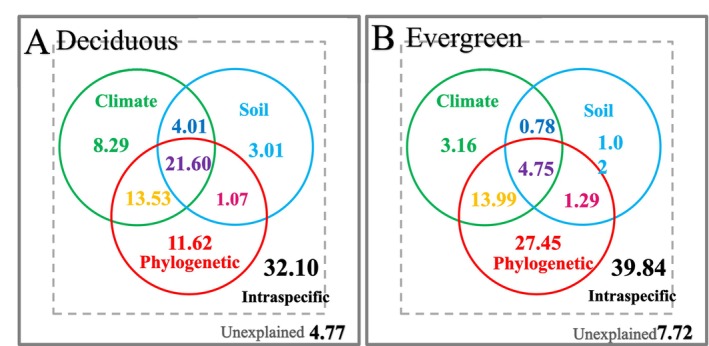
Relative contributions (%) of phylogenetic relatedness, intraspecific variation, climatic conditions, and soil properties to the variance in SLA in deciduous (A) and evergreen (B) woody angiosperms. Both PVR models included the interactive effects between climatic and soil variables on SLA.

The leaf phenology can influence these contributions. Within the woody angiosperms, some notable differences were found (Figure 7). The most profound difference was that the joint contribution of phylogenetic relatedness, soil properties, and climatic conditions was much higher in deciduous (21.60%) than that in evergreens (4.75%), making the individual contribution of phylogenetic relatedness much lower in deciduous (11.62%) than in evergreens (27.45%), although the total contribution of phylogenetic relatedness was similar between these two groups (47.82% and 47.48%, respectively).

### 3.5 Relative Contribution of Each Split to Tree‐Wide Variation in SLA

**FIGURE 8:**
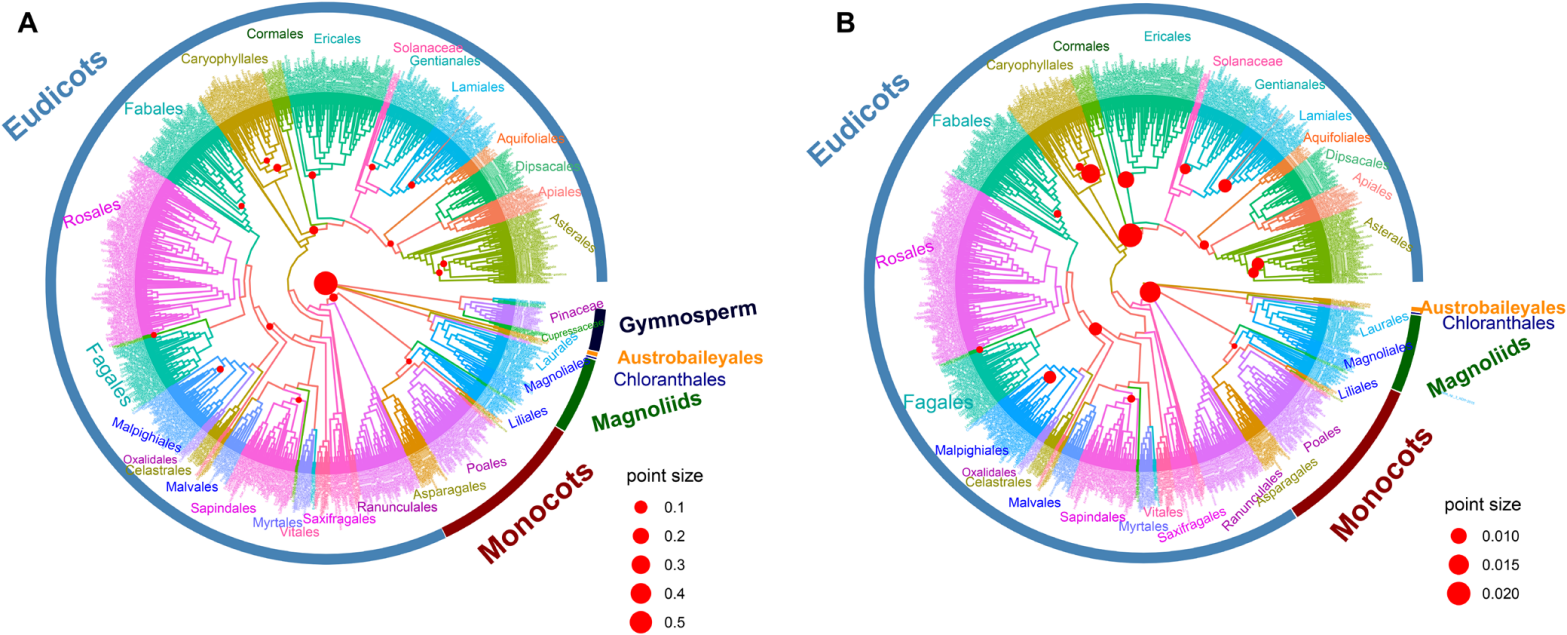
The relative contribution of each divergence node to tree‐wide variation in SLA. The relative contribution index (CI) is indicated by the size of the red circle. The branches of the same order are in the same color. The orders with more than 5 species were labeled. (A) The relative contribution of each divergence node along the phylogenetic tree. (B) The relative contribution of each divergence node along the phylogenetic tree but the gymnosperms are excluded.

The relative contribution index to tree‐wide variation (CI) was used to determine the importance of each split along the phylogenetic tree. The result showed that the split between angiosperms and gymnosperms (CI = 0.5842) had the largest contribution, whereas other splits had much less contribution (Figure 8, Table 2). The CI for the split between monocots and eudicots is 0.0160.

**TABLE 2:** The most important divergences in SLA. Clades involved in the 16 divergences that have a relative contribution index (CI) larger than 0.005.

Rank	CI	*D*	*p*	*n*	Age (myr)	Clades involved
1	0.584231	0.351	0.001	1264	325.050049	Angiosperm vs. Gymnosperms
2	0.020423	0.161	0.003	441	119.874252	Caryophyllales vs. Compositae, Apiales, Dipsacales, Aquifoliales, Lamiale, Gentianales, Solanaceae, Ericales, Cornales (in Rosidae)
3	0.016032	0.094	0.019	1168	135.758087	Monocots vs. Eudicots
4	0.013294	0.257	0.004	48	70.11264	Caryophyllaceae vs. Amaranthaceae (in Caryophyllales)
5	0.01068	0.485	0.001	102	103.577209	Balsaminaceae vs. Pentaphylacaceae, Theacea, Sapotaceae, Lecythidaceae, Ebenaceae, Primulaceae, Diapensiaceae, Styracaceae, Symplocaceae, Actinidiaceae, Clethraceae (in Ericales)
6	0.007736	0.699	0.004	32	52.9328	Plantaginaceae vs. Scrophulariaceae, Bignoniaceae, Acanthaceae, Paulowniaceae, Orobanchaceae, Lamiaceae (in Lamiales)
7	0.007472	0.367	0.003	399	116.810913	Zygophyllales vs. Celastrales, Oxalidales, Malpighiales, Fagales, Cucurbitales, Rosales, Fabales (in Rosidae)
8	0.007204	0.336	0.002	22	94.362061	Violaceae vs. Salicaceae (in Malpighiales)
9	0.006992	0.223	0.01	43	21.448792	*Petasites, Ligularia, Parasenecio, Syneilesis, Crassocephalum, Senecio* vs. *Tanacetum, Achillea, Hippolytia, Ajania, Chrysanthemum, Pyrethrum, Seriphidium, Artemisia, Aster, Heteropappus, Asterothamnus* (In Compositae)
10	0.006134	0.221	0.015	50	22.989471	*Inula, Karelinia, Anaphalis, Xanthium* vs. *Astrothamnus, Heteropappus, Aster, Artemisia, Seriphiadium, Pyrethrum, Chrysanthemum, Ajiania, Hippolytia, Achillea, Tanacetum, Senecio, Crassocephalum, Parasenecio, Syneilesis, Ligularia, Petasites* (In Compositae)
11	0.00613	0.186	0.046	57	85.913147	Solanales vs. Gentianales (in Rosidae)
12	0.005599	0.166	0.008	144	102.692856	Aquifoliales vs. Dipsacales, Apiales, Asterals (in Rosidae)
13	0.005243	0.182	0.005	41	40.249451	*Agriophyllum, Corispermum, Ceratocarpus, Krascheninnikovia, Axyris, Dysphania, Chenopodium, Atriplex* vs. *Halostachys, Kalidum, Salicornia, Suaeda, Nanophyton, Petrosimonnia, Sympegma, Anabasis, Haloxylon, Horaninovia, Halogeton, Salsola, Kochia, Bassia* (in Amaranthaceae)
14	0.005215	0.329	0.014	61	83.350235	Liliales vs. Asparagales, Poales, Zingiberales, Commelinales and Arecales (in Monocots)
15	0.005178	0.434	0.043	54	63.513794	*Amorpha* vs. *Indigofera, Millettia, Tephrosia, Fordia, Cajianus, Campylotropis, Kummerowia, Lespedeza, Dendrolobium, Hylodesmum, Pueraria, Robinia, Clycyrrhiza, Callerya, Wisteria, Caragana, Alhagi, Hedysarum, Onobrychis, Astrafalus, Vicia, Melilotus, Medicago, Trifolium* (in Fabaceae)
16	0.005073	0.595	0.002	4	56.532288	Begoniaceae vs. Coriariaceae (in Cucurbitales)

Abbreviations: Age, age of divergence; *D*, divergence size; *n*, number of species involved; *p*, *p*‐value for the divergence size.

Some large CIs were found among orders. For instance, within the Rosidae, the split between Caryophyllales and the clade consisting of Compositae, Apiales, Dipsacales, Aquifoliales, Lamiale, Gentianales, Solanaceae, Ericales, and Cornales exhibited a CI of 0.02423, and the split between Zygophyllales and the clade consisting of Celastrales, Oxalidales, Malpighiales, Fagales, Cucurbitales, Rosales, and Fabales had a CI of 0.0075.

Important splits were also found between families within Caryophyllales (CI = 0.0133), Ericales (CI = 0.0107), Lamiales (CI = 0.0077), Malpighiales (CI = 0.0072), and Cucurbitales (CI = 0.0051). Similarly, there were important splits between genera within Compositae (CI = 0.0070), Fabaceae (CI = 0.0052), and Amaranthaceae (CI = 0.0053, Table 2).

## 4 Discussion

### 4.1 The Importance of Phylogenetic Relatedness

Significant phylogenetic signals of specific leaf area (SLA) have been detected regionally and globally (Flores et al. 2014; Liu, Zhao, et al. 2022; Albassatneh et al. 2023). Our results also showed significant phylogenetic signals of SLA in China, suggesting that closely related species had more similar SLA, that is, the phenomenon of phylogenetically niche conservatism (Lohbeck et al. 2015; Zhang et al. 2019; Akram et al. 2022).

A previous study implied that the most important factors to the variation in SLA across China were the environmental conditions rather than the phylogeny (An et al. 2021). However, our results suggested that it was the phylogeny explaining the majority of the variance (58%) in SLA across China. This difference may be mainly derived from the different analytic methods. An et al. (2021) partitioned the total variance in SLA by a phylogenetic linear mixed model (PLMM). They considered the influence of phylogeny as random effects in the model and set the random term as family nested in order and order nested in clade (clade/order/family). Therefore, the variation within family was treated as residual errors. However, the variation in SLA within certain families (e.g., Fabaceae) or genera (e.g., *Dendrobium*) was also strongly affected by the phylogeny (Sun et al. 2020; Liu et al. 2024). Consistent with these studies, our results showed that the interspecific variation within family accounted for 25% of the total variance in SLA, while the inter‐family variation within phylum only accounted for 15% of the total variance (Figure 3B). Therefore, we used the phylogenetic eigenvector regression (PVR), which could well incorporate the interspecific variation, to estimate the relative importance of phylogeny.

Another difference between PLMM and PVR was that the exact phylogenetic information used in PLMM was the phylogenetic tree topology, which ignored the explicit branch length and thus did not consider the differential evolutionary distances among phylogenetic families (An et al. 2021). The evolutionary rates were not constant along the entire phylogenetic tree and could vary among different clades (Shao et al. 2019). For instance, younger clades might have greater diversification rates than older ones if there is a diversification‐declining trend (Chen 2013; Lu et al. 2018). The PVR used in this study was a purely data‐driving method (i.e., independent of any evolutionary model) that can flexibly relate the phylogeny to plant traits (Seger et al. 2012). Therefore, the relative importance of evolutionary history to the variation in SLA across China could be much larger than previously considered.

### 4.2 Differential Effects of Environmental Variables on SLA Between Gymnosperms and Angiosperms

Previous studies have demonstrated that climatic conditions and soil properties could influence SLA (Wright et al. 2005; Madani et al. 2018; Ahrens et al. 2020; Xing et al. 2021). However, the distinctive effects of these environmental variables on SLA between gymnosperms and angiosperms remain unclear. We found that MAT, MTCM, and MTHM significantly affect the SLA of gymnosperms but had weak influences on the SLA of angiosperms (Figure 4B–D). The increased SLA with MAT and MTHM in gymnosperms might be due to the decreases in leaf thickness and cell layers and the increase in cell size with the increasing temperatures (Rosbakh et al. 2015). The SLA of gymnosperms exhibited the lowest value when the MTCM was modest (Figure 4C). The large SLA in sites with MTCM of ~ − 30°C was due to the trees ( *Larix kaempferi* and *Larix gmelinii* ) being deciduous. Indeed, the SLA of deciduous gymnosperms (154.23 ± 54.73, mean ± SD) was larger than that of evergreen ones (84.87 ± 35.12) when the MTCM was below −15°C, which was consistent with a global comparison between the deciduous and evergreen trees (Wright et al. 2005). The weak effects of temperature on SLA in angiosperms could be the result of the extremely high diversity in this plant taxa. Angiosperms were the most complex taxon in Spermatophyta and the most‐diverse group within the Plantae (Benton et al. 2022). There was a nearly 200‐fold variation in the SLA within angiosperms (Xing et al. 2021). Along a gradient of decreasing MAT, SLA decreased in evergreens but increased in deciduous species (Wright et al. 2005). Liu, Wang, et al. (2017) found that the SLA of *Stipa* was positively associated with MAT, while those of *Cleistogenes* and *Carex* showed the opposite pattern in northern China grassland (Liu, Wang, et al. 2017). We also found that within woody angiosperms, the evergreens had significant but weak relationships with thermal factors, whereas the deciduous showed non‐significant relationships (Figure 5B–D).

Both gymnosperms and angiosperms SLA were strongly correlated with water conditions with similar trends, which were generally consistent with previous studies (Wright et al. 2005; Meng et al. 2015; Gong et al. 2020). With the increases in precipitation and moisture index (MI), the SLA first increased and then decreased (Figure 4E,F). Plants tend to keep smaller and thicker leaves to reduce water loss in arid sites and have larger and thinner leaves in water‐rich environments to promote light sequestration and enhance competitive capacities (Liu, Wang, et al. 2017). The decreased SLA in sites with high MAP or MI might be derived from several reasons. First, the sites with extremely high precipitation also had high potential evapotranspiration (PET), leading to reduced water availability for plants (Niu et al. 2019). Second, the wetter sites also had higher solar radiation with a greater amount of UV‐B. UV‐B may induce smaller SLA by thickening leaves and reducing leaf area, which morphological changes can reduce the cell layers that are exposed to ambient UV‐B (Jansen 2002; Robson and Aphalo 2012; Robson et al. 2015). Third, heavy rainfalls in wet regions could cause stronger weathering and leaching, resulting in the loss of clay particles and nutrients (Libalah et al. 2017), which will constrain the SLA (Poorter et al. 2009). Fourth, plants are at high risk from phytophagous animals in wet regions (Anstett et al. 2016). The low SLA in these regions can better defend against herbivores and physical hazards (Baruch 2011).

For the effects of soil properties, a relatively strong effect was only found for the influence of soil total nitrogen content (TN) on SLA in angiosperms (Figure 4K). In the habitat with poor nutritional conditions, the plants embraced the strategy of reducing SLA to improve stress resistance (Poorter et al. 2009; Zhou et al. 2020). In the habitat with rich nutritional conditions, greater SLA was beneficial to enhancing the photosynthetic capacity and productivity, thus strengthening the competitive ability among species (Yao et al. 2016; Zhou et al. 2020). On the contrary, there was no significant relationship between TN and SLA within gymnosperms (Figure 4K). This was probably due to the large proportion of ectomycorrhizal (ECM) plants within the gymnosperms in our dataset. In the dataset, 75.25% of species belonged to Pinaceae, which family was ectomycorrhizal‐obligated (Hayward et al. 2015). ECM fungi can break down soil organic matter (SOM) by producing hydrolytic and oxidative extracellular enzymes, which enable these fungi to mine soils for compounds that contain nitrogen in order to release nutritional constraints in ECM plants (Phillips et al. 2013; Zak et al. 2019). As a result, the variation in SLA was relatively independent of the soil nitrogen within gymnosperms.

### 4.3 The Effects of Leaf Longevity

Both leaf longevity and SLA are important leaf economics traits (Wright et al. 2004, 2005). In this study, we compared the differential effects of climate and soil on SLA between evergreen and deciduous woody angiosperms. The most profound result was that the SLA of deciduous species was generally higher than that of evergreen ones under the same environmental conditions (Figure 5). Evergreen species have a longer leaf longevity than the deciduous ones, reflecting a trade‐off along the leaf economics spectrum: longer leaf longevity is linked to lower SLA and slower growth rates, and short‐lived leaves prioritize high SLA for rapid carbon return (Givnish 2002; Reich 2014).

Leaf longevity also regulated the effects of temperature, precipitation seasonality, and soil N and P contents on the SLA (Figure 5B–D,H,K,L). Temperature significantly increased the SLA in evergreen species but not in deciduous species, which might reflect the complicated effects of temperature. On one hand, higher temperature was associated with faster plant growth, which required higher SLA (Osone et al. 2008; Ganjurjav et al. 2022). On the other hand, higher temperature caused a longer growing season, resulting in a longer leaf lifespan for deciduous species and thus may be related to a smaller SLA (Kikuzawa et al. 2013). Within deciduous species, the effect of one mechanism may offset the other, resulting in a non‐significant trend. In fact, some studies even found a negative relationship between SLA and temperature in deciduous species (Wright et al. 2005).

When the precipitation seasonality was relatively high, the SLA decreased with precipitation seasonality within both evergreen and deciduous species (Figure 5H). When the total annual precipitation kept the same, higher precipitation seasonality means that more rainfall is concentrated in a specific period (Kelley et al. 2013). Excessive water in a short period actually reduced the total available water for plants and caused drought conditions in the long term (Bodner et al. 2015). To cope with the drought stress, plants tended to enhance their leaf longevity and thus decreased the SLA (Kaproth et al. 2023). Such an effect was weaker in deciduous than in evergreen species because deciduous plants can drop their leaves to avoid drought (Singh and Kushwaha 2016), which mechanism limited their leaf longevity.

SLA in evergreens showed a clear trend that first increased and then decreased with the increase in TN, which is inconsistent with previous studies (Wright et al. 2005; He et al. 2018). The declining trend might be due to the sites with TN > 200 g kg^−1^ having low MAT (MAT < 12°C), which decreased the SLA. The effect of soil P on SLA in deciduous species was similar to that in the angiosperms, whereas that in evergreen species was negative (Figure 5L). This probably reflected the influences of other factors as the TP was negatively correlated with both MAT (*r* = −0.52, *p* < 0.001) and MAP (*r* = −0.54, *p* < 0.001). Nevertheless, the exact reasons need to be further examined. Overall, incorporating the influences of leaf longevity can improve the explanatory power of climate and soil factors to explain the variation in SLA by 3%–11% within the woody angiosperms.

### 4.4 Relative Contribution of Divergence Node to Tree‐Wide Variation

Besides the difference between gymnosperms and angiosperms, SLA also exhibited significant differences between many clades along the phylogenetic tree. For example, the SLA of monocots (226 cm^2^ g^−1^) was larger than that of eudicots (201 cm^2^ g^−1^). The divergence among monocots and eudicots in angiosperm evolution was quite early (between 140 and 150 Mya; Hertweck et al. 2015; Coiro et al. 2019), resulting in great diversity in leaf morphologies in both phylogenetic clades (Conklin et al. 2019). For example, the leaves can vary from dorsoventrally flattened leaves to dissected leaves with broad lamina and petioles and to cylinder‐shaped, unifacial leaves (Conklin et al. 2019). Compared to monocot leaves, the eudicot leaves usually have a distinct petiole attaching the leaf blade to the stem, allowing the leaf to move and adjust its position to maximize sunlight exposure (Leeflang et al. 1998). The strong light exposure was usually associated with small SLA (Liang et al. 2020; Du et al. 2021). Besides, monocot leaves may have distributed, parallel rows of stomata while the stomata of eudicots are randomly distributed across the epidermis (Hepworth et al. 2018; Conklin et al. 2019). These differences in stomatal traits may also result in differential SLA (Wang et al. 2016b; Du et al. 2021).

The contribution of difference within Rosidae to the total variance in SLA was the second largest (Table 2). Rosidae originated in the early to late Cretaceous (115 to 93 Mya) and experienced nested radiative evolution. This family consists of approximately 90,000 angiosperms, with remarkable diversity of morphologies and extraordinary heterogeneity in habitats and life forms (Sun et al. 2016, 2020). For example, plants in Rosidae exhibit various life forms such as trees, shrubs, vines, herbs, succulents, parasites, and aquatics (Sun et al. 2016) and occupy most terrestrial habitats, including arctic, alpine, desert, temperate, and tropical areas (Sun et al. 2020). Within the Rosidae, the SLA in Caryophyllales (116.15 cm^2^ g^−1^) was smaller than that in the clade consisting of Compositae, Apiales, Dipsacales, Aquifoliales, Lamiales, Gentianales, Solanaceae, Ericales, and Cornales (221.02 cm^2^ g^−1^, Table 2) probably due to the small SLA of succulence and C4 or CAM plants in Caryophyllales (Cornwell et al. 2014).

At the order level, species within the Caryophyllales exhibited a large diversity of life‐history, ranging from temperate annual herbs to tropical trees, and from succulents to carnivorous plants (Smith et al. 2018). Ericales and Lamiales also exhibited large variation in SLA, which might be attributed to the rapid radiative evolutions in these two orders during the past million of years (Rose et al. 2018).

At the family level, Compositae is the largest family of flowering plants including more than 25,000 species (Funk et al. 2009; Zhang and Elomaa 2024). This family originated in South America at least 50–70 Mya, and distributes cosmopolitanly (Funk et al. 2009). Plants in Compositae adapt to extensive environments and habitats through several mechanisms such as producing abundant and diverse secondary metabolites to prevent herbivory and possessing a special dual‐purpose fruit structure that both prevents herbivory and promotes long‐distance seed dissemination (Funk et al. 2009; Mandel et al. 2017). Root nodule symbiosis between Fabaceae family plants and rhizobia, soil bacteria that fix nitrogen, lowers the requirement for external nitrogen (Yang et al. 2022), making the Fabaceae exhibit a wide range of growth forms from annual herbs through lianas to shrubs and tall trees (Yahara et al. 2013). As a result, the SLA in Fabaceae has a great variation (45–327 cm^2^ g^−1^).

### 4.5 Uncertainties and Conclusions

Some uncertainties might exist. For a certain species, it is well known that the SLA values can be influenced by plant and leaf ages, light intensity, and sampling season (Kitajima 2002; Wright et al. 2004; Jagodziński et al. 2016). Fortunately, the data used in our study was from the standardized protocol that leaf samples were collected from adult plants, prioritizing young, healthy, and fully expanded leaves with hardened laminar structures. Outer canopy leaves from individuals under optimal growth conditions are preferred (Pérez‐Harguindeguy et al. 2013). The influence of sampling seasons was more difficult to eliminate, especially for the data collected by the same research group. Nevertheless, studies aimed to estimate the species functional traits would sample the leaves in their growing season. For the dataset that had heterogeneous sources (e.g., TRY Plant Trait Database, https://www.try‐db.org), some data cleaning processes might be necessary to minimize the sampling errors.

China covered a vast area with diverse climatic types (Xu et al. 2023; Ge et al. 2023), some of which may be extremely sensitive to climate change but have a very low sample size (e.g., the Tibetan Plateau and the Xing'an Mountains region, Liu et al. 2015; Seddon et al. 2016; Xu et al. 2024). More experimental work in these areas will undoubtedly facilitate studies on the influence of evolutionary history on plant functional traits since plant evolutionary processes could be especially unique in such stressed areas (Liu, Luo, et al. 2017; Jin et al. 2023).

In conclusion, we underscored the importance of evolutionary history in shaping the variation in SLA across China, with the relative importance being 50.46%, substantially larger than those of climate and soil conditions. As we have mentioned, our calculation based on the PVR method will extract the variation derived from the phylogenetic relatedness as much as it can; therefore, exhibiting a much larger contribution of evolutionary history than a previous study used a similar dataset (An et al. 2021).

Along the phylogenetic tree, the most distinctive difference (60%) occurred at the divergence of gymnosperms and angiosperms. Indeed, these two clades received much attention and reflected very fundamental differences in plant morphology and physiology (Wright et al. 2005; Wang et al. 2016a). Fewer studies focused on finer taxa (e.g., Schwery et al. 2015 for Ericaceae). However, such detailed information could as well be important consideringthat the remaining variation in SLA (40%) was also large and there was high heterogeneity in the evolutionary rates (Flores et al. 2014). More importantly, we found differential effects of climate and soil conditions on the SLA between gymnosperms and angiosperms and a relatively high joint contribution of climate, soil, and phylogeny within the deciduous woody angiosperms (Figures 4 and 7A), highlighting the important role of the potential interaction between the external environment and the phylogeny. However, how to reveal such potential interactions along phylogenetic trees with heterogeneous evolutionary rates remains a challenging task (Li et al. 2024). These efforts could be extremely helpful for incorporating the evolutionary information in ecosystem models, thus improving the accuracy of model predictions on ecosystem functions in a rapidly changing world.

## Author Contributions

**Minyue Si:** conceptualization (lead), formal analysis (lead), visualization (lead), writing – original draft (lead), writing – review and editing (lead). **Caiyi Zhang:** writing – review and editing (supporting). **Chunzhu Xiang:** formal analysis (supporting). **Mingxia Jiang:** formal analysis (supporting). **Linwei Guo:** formal analysis (supporting). **Junjiong Shao:** conceptualization (supporting), writing – review and editing (lead).

## Conflicts of Interest

The authors declare no conflicts of interest.

## Supporting information


Data S1

Figure S1

## Data Availability

The data that supports the findings of this study are available in the Supporting Information of this article (Data S1). The main data was extracted from the China Plant Trait Database version 2 (CPTDv2): https://doi.org/10.1038/s41597‐022‐01884‐4 (Wang et al. 2022).
