# Supplementary material for: The Role of Plant Evolutionary History in Shaping the Variation in Specific Leaf Area Across China
Source: Ecol Evol. 2025 Apr 18;15(4):e71304. doi: 10.1002/ece3.71304 (PMC12008053; doi:10.1002/ece3.71304)
Supplement: Supplementary file 2 — Figure S1 [file ECE3-15-e71304-s001.docx]

Fig S1 Influences of topsoil sand fraction (A), topsoil silt fraction (B), topsoil clay fraction (C) and topsoil cation exchange capacity (D) on SLA of gymnosperms and angiosperms. Blue and purple colors represent the angiosperms and gymnosperms, respectively. Regression lines are based on the linear mixed-effects models.
